# Male obesity is associated with sperm telomere shortening and aberrant mRNA expression of autophagy-related genes

**DOI:** 10.1186/s12610-023-00188-w

**Published:** 2023-05-25

**Authors:** Pourya Raee, Zahra Shams Mofarahe, Hamid Nazarian, Mohammad-Amin Abdollahifar, Mahsa Ghaffari Novin, Shahin Aghamiri, Marefat Ghaffari Novin

**Affiliations:** 1 Department of Biology and Anatomical Sciences, School of Medicine, Shahid Beheshti University of Medical Sciences, Tehran, Iran; 2 Department of Biotechnology, School of Advanced Technologies in Medicine, Shahid Beheshti University of Medical Sciences, Tehran, Iran

**Keywords:** Obesity, Semen analysis, DNA fragmentation, Telomere, Autophagy, Reactive oxygen species, Obésité, Analyse du Sperme, Fragmentation de l’ADN, Télomère, Autophagie, Espèces réactives de l’Oxygène

## Abstract

**Background:**

Obesity is regarded a global public health crisis. It has been implicated in a variety of health problems, but when it comes to male fertility, how and to what extent obesity affects it are poorly understood.

Accordingly, semen samples from 32 individuals with obesity (body mass index (BMI) ≥ 30 kg/m^2^) and 32 individuals with normal weight (BMI: 18.5-25 kg/m^2^) were obtained. Here, for the first time, we examined the association between obesity, relative sperm telomere length (STL) and autophagy-related mRNA levels such as Beclin1, AMPKa1, ULK1, BAX, and BCL2. Each group was also evaluated for conventional semen parameters, sperm apoptotic changes, DNA fragmentation index (DFI), sperm chromatin maturation, and reactive oxygen species (ROS) levels.

**Results:**

Based on our findings, there was a marked reduction in relative STL in individuals with obesity as compared to the normal-weight group. We also found a significant negative correlation between relative STL and age, BMI, DFI, percentage of sperm with immature chromatin, and intracellular ROS levels in patients with obesity. In the normal-weight group, relative STL was only negatively correlated with DFI and intracellular ROS levels. Regarding mRNA expression, there was considerable upregulation of Beclin1, ULK1, and BCL2 in the group with obesity compared to the normal-weight group. Obesity was also found to be associated with a considerable decline in semen volume, total sperm count, progressive motility, and viability in comparison to normal-weight individuals. Furthermore, obesity was associated with considerably higher percentages of DFI, sperm with immature chromatin, late-stage apoptosis, and elevated ROS levels.

**Conclusion:**

According to our findings, obesity is associated with sperm telomere shortening and aberrant autophagy-related mRNA expression. It should be emphasized that telomere shortening in sperm may be an indirect consequence of obesity due to the oxidative stress associated with the condition. Nevertheless, further investigation is required for a more comprehensive understanding.

## Résumé

**Contexte:**

L’obésité est considérée comme une crise mondiale de santé publique. Elle a été impliquée dans divers problèmes de santé ; mais quand il s’agit de la fertilité masculine, comment et dans quelle mesure l’obésité affecte cette fertilité restent mal compris. En conséquence, des échantillons de sperme de 32 hommes obèses (indice de masse corporelle (IMC) ≥ 30 kg/m²) et de 32 hommes ayant un poids normal (IMC : 18,5 à 25 kg/m²) ont été recueillis. A été examiné dans cette étude, pour la première fois, l’association entre l’obésité, la longueur relative des télomères des spermatozoïdes (LTS), et les taux d’ARNm liés à l’autophagie tels que Beclin1, AMPKa1, ULK1, BAX et BCL2. Chaque groupe a également été évalué pour les paramètres conventionnels du sperme, les changements apoptotiques des spermatozoïdes, l’indice de fragmentation de l’ADN (DFI), la maturation de la chromatine des spermatozoïdes et les niveaux d’espèces réactives de l’oxygène (ROS).

**Résultats:**

Il y eut une réduction marquée de la LTS relative chez les hommes obèses par rapport à ceux du groupe de poids normal. Nous avons également trouvé une corrélation négative significative entre la LTS relative et l’âge, l’IMC, le DFI, le pourcentage de spermatozoïdes avec chromatine immature et les niveaux intracellulaires de ROS chez les hommes obèses. Dans le groupe d’hommes de poids normal, la LTS relative n’était corrélée négativement qu’avec les taux de DFI et de ROS intracellulaires. En ce qui concerne l’expression de l’ARNm, il y avait une régulation positive considérable de Beclin1, ULK1 et BCL2 dans le groupe d’hommes obèses par rapport à ceux du  groupe de poids normal. L’obésité s’est également avérée être associée à une baisse considérable du volume de sperme, du nombre total de spermatozoïdes, de la mobilité progressive et de la viabilité des spermatozoïdes par rapport aux hommes de poids normal. En outre, l’obésité était associée à des pourcentages considérablement plus élevés de DFI, de spermatozoïdes avec chromatine immature, d’apoptose à un stade avancé, et de niveaux élevés de ROS.

**Conclusion:**

Selon nos résultats, l’obésité est associée au raccourcissement des télomères des spermatozoïdes et à une expression aberrante d’ARNm liés à l’autophagie. Il convient de souligner que le raccourcissement des télomères dans les spermatozoïdes peut être une conséquence indirecte de l’obésité en raison du stress oxydatif associé à la maladie. Néanmoins, des études plus approfondies sont nécessaires pour une compréhension plus complète.

## Background

Over the past few decades, obesity has become one of the fastest-growing health issues in the world, to the point where it has been considered an epidemic health crisis. Obesity is described by the World Health Organization (WHO) as an excessive deposition of fat that may have a negative impact on health. It is characterized as a body mass index (BMI) of 30 kg/m^2^ or more [1, 2]. In 2016, according to the WHO, 11.1% of men over the age of 18 showed obesity, while 38.5% were overweight, implying that nearly half of the male population worldwide are individuals with overweight or obesity [3].

It has been known that obesity can affect male fertility through several different mechanisms. Steroidogenesis and spermatogenesis are both negatively associated with increased BMI, which is generally linked to high visceral adiposity. Thus, the aromatization of steroids to estrogens in the peripheral adipose tissue results in elevated levels of estradiol, which exerts negative feedback at the hypothalamic-pituitary level, leading to secondary hypogonadism. Inflammation and oxidative stress can also have consequences at the local level by altering spermatogenesis, increasing sperm DNA fragmentation, and leading to germ cell apoptosis [4, 5]. Oxidative stress may play a critical role in sperm alteration associated to obesity [6, 7]. Excessive production of reactive oxygen species (ROS) combined with the marked decline in antioxidant capacity can lead to oxidative stress (OS). This imbalance has a significant impact on second messengers and intracellular signaling pathways, resulting in deleterious effects on the tissue or cell function (including spermatozoa), as well as direct damage to cell components (proteins, lipids, and DNA) [6, 7].

Apart from these mentioned mechanisms, there is a growing need for more in-depth knowledge of how and to what extent obesity is associated with sperm parameters. As such, knowing the underlying mechanisms would present us the opportunity to take preventative or therapeutic measures against obesity-induced subfertility across the male population.

A further potential avenue in this context is to explore alterations in the sperm telomere length (STL) and autophagy-related mRNA expression. Telomeres are repeating nucleotide sequences found at the ends of chromosomes, and their length is regarded as a biomarker for DNA integrity [8]. Telomerase, a specialized reverse transcriptase, is highly expressed in germ cells and serves as an architect to ensure that telomere length is maintained [8–10]. Obesity has been demonstrated to be associated with reduced telomere length. For instance, a recent meta-analysis showed that obesity is associated with leukocyte telomere length shortening [11]. This can be one of the main reasons for investigating the possible relationship between obesity and STL alteration. The lesser-known role of sperm telomeres in obesity-related alteration of semen parameters complicates the matter even more. Multiple studies have investigated STL in the context of different aspects of male fertility/infertility [9, 10, 12]. There is only one study that included overweight/obese males (BMI > 28 kg/m2) [13]. The authors included a large number of patients (overweight/obese males (*n* = 306), and normal-weight males (345)). They observed that overweight/obese males had a shorter sperm telomere length than normal-weight individuals [13]. In terms of STL, however, no studies have been conducted purely on obesity (BMI ≥ 30 kg/m^2^). Aside from that, the present study has a number of fundamental differences from the mentioned study [13], which will be discussed in detail in the discussion section.

Dysregulation of autophagy is another potential candidate playing a role in obesity-related alteration of sperm parameters [14–16]. It has been found that the autophagy mechanism is actively present in spermatozoa and can be dysregulated through diet-induced obesity in mouse models [15, 16]. Autophagy is essential and advantageous to cells as it eliminates damaged organelles and supplies bioenergetic substrates required for cell survival [17]. It is critical to understand that over-activation of autophagy does not promote cell survival but rather causes cell death and reduces viability [17]. In the case of obesity, cells are deprived of energy substrate, so the AMP-activated protein kinase (AMPK) signaling pathway begins to trigger autophagy in response to nutrient stress, namely the bio-availability of amino acids and glucose, or any other forms of stress. In addition to the mammalian target of rapamycin complex 1 (mTORC1) inhibition, AMPK can directly phosphorylate and activate Unc-51-like kinase 1 (ULK1) to promote autophagy. A further step in the autophagy pathway is initiated when the ULK1 complex interacts with vacuolar protein sorting 34 (VPS34) and forms complexes with key autophagy elements, such as Beclin1 [14, 17]. Meanwhile, B-cell lymphoma 2 (BCL2) can establish a complex with Beclin1, inhibiting autophagy mediated by this protein. In addition, BCL2-associated X protein (BAX) is intimately related to BCL2, so understanding the involvement of BAX as an inducer of apoptosis seems crucial [18, 19]. Based on the abovementioned points, assessing the expression of these genes could give us viable information about the association of obesity with the autophagy process.

Based on what has been described so far, it appears necessary to examine the relationship between STL and variables such as sperm DNA integrity, apoptotic changes, chromatin maturation, ROS levels, and autophagy-related mRNA expression in the semen samples of individual with obesity as one of the possible underlying conditions in obesity-related sperm alteration. With this in mind, our purpose was to examine the association between obesity, relative STL, mRNA expression of autophagy-related genes (Beclin1, AMPKa1, ULK1, BAX, and BCL2), values of semen parameters, sperm DNA integrity, chromatin maturation, apoptotic changes, and intracellular ROS levels.

## Materials and methods

### Participants

Semen samples were collected through masturbation from 32 individuals with obesity (BMI ≥ 30 kg/m^2^) (*n* = 32) and 32 normal-weight (BMI: 18.5-25 kg/m^2^) men (*n* = 32) referring to Taleghani Hospital, Tehran, Iran. We recruited patients from the general population who presented to the fertility clinic for a routine fertility evaluation before trying to conceive. Each participant signed an informed consent form. All participants were under the age of 45 years. In addition, there were no indications of genital infection, Klinefelter’s syndrome, hypogonadism, anatomical disorders, varicocele, azoospermia, scrotal trauma or surgery, diabetes, smoking, alcohol consumption, exposure to pesticides and solvents, and/or any history of receiving weight loss or cholesterol-lowering medications (Orlistat, Atorvastatin, and Gemfibrozil). Note that at the beginning of the study, a total of 72 patients were enrolled (35 individuals with obesity and 37 normal-weight individuals). However, one patient from the group with obesity and two patients from the normal-weight group later declined to participate in the study. Furthermore, two patients from the group with obesity and three patients from the normal-weight group were excluded from the study based on the previously indicated exclusion criteria.

## Sample size calculation

For the sample size calculation, we used the following formula [20]:

$$\mathrm{Sample size}=\frac{r+1}{\mathrm{r}}\frac{{{ {\mathrm{SD}}^{2}(z}_{\beta }+{z}_{\alpha /2})}^{2} }{{\mathrm{d}}^{2}}=\frac{1+1}{1} \frac{{{0.3}^{2}(1.28 +1.96 )}^{2} }{{(0.25)}^{2}}=30.234$$

SD = Standard deviation.

d = Expected mean difference between two groups.

r = Ratio of control to cases.

Zβ = Standard normal variate for power.

Za/2 = Standard normal variate for the level of significance.

### Sperm preparation

After 3 to 5 days of abstinence from sexual activity, semen samples were obtained from all participants and liquified for 20 min at 37 °C. Semen analysis was carried out in accordance with the protocol established by the WHO [21]. Total sperm count, sperm concentration, morphology, and motility were all assessed using an aliquot of the semen sample. The remainder of each sample was then used to evaluate sperm DNA fragmentation index (DFI), chromatin maturation, viability/apoptosis, and intracellular ROS levels via sperm chromatin dispersion (SCD) test, aniline blue staining, annexin V-FITC/PI staining, and 2'-7'dichlorofluorescin diacetate (DCFH-DA) assay, respectively. The relative STL and the mRNA level of Beclin1, AMPKa1, ULK1, BAX, and BCL2 were also assessed utilizing quantitative real-time PCR (qRT-PCR).

### Sperm DNA fragmentation index (DFI)

In order to assess DFI, SCD test was performed utilizing the SDFA kit (IVF, Tehran, Iran), as instructed by the manufacturer. This assay relies on measuring the dispersion of nuclear chromatin after the removal of nuclear proteins and DNA denaturation. In sperms with fragmented DNA, the peripheral halo of the dispersed DNA loop is not evident, whereas it is clearly visible in normal sperm with intact DNA. Knowing this, 300 spermatozoa per each sample were observed at 1000 × magnification using a bright-field microscope. The proportion of sperm with fragmented DNA was then recorded [22].

### Sperm chromatin maturation assay (aniline blue (AB) staining)

After air-drying a fresh sperm smear from each sample, each smear was fixed and then stained using SCMA kit (IVF, Tehran, Iran), as instructed by the manufacturer. Finally, 200 spermatozoa per each stained sample were examined under oil immersion and 1000 × magnification using a bright-field microscope. Next, the percentage of sperm with immature chromatin (blue-stained nuclei) was recorded [23, 24].

### Evaluation of intracellular ROS levels

DCFH-DA, a cell-permeant reagent, is a fluorogenic dye that detects ROS activity within the cell. Once this reagent is taken up by cells, cellular esterases convert it to a nonfluorescent molecule, which is then oxidized by ROS, producing a fluorescent dye with maximal excitation and emission wavelengths of 488 and 535 nm, respectively. To measure ROS levels, sperm cells were washed and treated for 20 min at 37 C with phosphate buffer saline (PBS) containing 25 μM DCFH-DA, followed by 10 min of incubation at 37 C in a 12 μM propidium iodide (PI) solution to distinguish live from dead cells. Finally, the mean fluorescent intensity (MFI) of DCF in live sperm cells was determined using flow cytometry [25].

### Evaluation of viability and apoptotic changes using annexin V-FITC/PI staining

Semen samples were tested for viable, apoptotic (early and late), and necrotic sperm using an Annexin V/PI binding Assay (Abcam, Cambridge, UK). Briefly, each semen sample was double washed using PBS. Next, 1 × 10^6^ spermatozoa were resuspended in 1 ml of 1X Annexin V binding buffer. Each sample was then stained by gently mixing 5 μl of Annexin V-FITC and 5 μl of PI into the sample followed by incubation at 25° C for 15 min in the darkness. Finally, the percentages of live, early apoptotic, late apoptotic, and necrotic sperm were determined through flow cytometry.

### Sperm telomere length (STL) assay by q-PCR

The QIAamp DNA Mini Kit (QIAGEN, Milan, Italy) was used to isolate DNA from the washed sperm samples. RealQ plus 2 × Master Mix Green (Ampliqon, Denmark) was utilized for qRT-PCR. All samples were run in triplicate, and the relative STL was calculated in the manner stated earlier [26, 27]. Briefly, the ratio of telomeres to single-copy genes was used to calculate the relative STL, and the result was reported as a fold change (2^(−ΔΔct)^). Telomere and single-copy gene (beta-globin) primer pairs are listed in **Table ****1**.

**Table 1:** Characteristics of designed primers

**STL assay**	**Genes**	**Forward primer (5' –3')**	**Reverse primer (5'–3’)**
	**Telomere**	TelG: ACACTAAGGTTTGGGTTT GGGTTTGGGTTTGGGTTAGTGT	TelC: TGTTAGGTATCCCTATCCC TATCCCTATCCCTATCCCTAACA
	**Beta-globin** **(Single-copy gene)**	hbgu: CGGCGGCGGGCGGCGCG GGCTGGGCGGcttcatccacgttcaccttg	hbgd: GCCCGGCCCGCCGCGCC CGTCCCGCCGgaggagaagtctgccgtt
**mRNA expression**	**Beclin1**	CAAGATCCTGGACCGTGTCA	GGCACTTTCTGTGGACATCATC
	**AMPKa1(PRKAA1)**	ACAGCCGAGAAGCAGAAACAC	TCATGTGTGCCAACCTTCACT
	**ULK1**	CCGCGAGAAGCACGATTTG	AGTCATACAGGGCCACGATG
	**BAX**	GAGCAGATTATGAAGACAGGGG	ACGGCGGCAATCATCCTC
	**BCL2**	AGATTGATGGGATCGTTGCCT	AGTCTACTTGCTCTGTGATGTTGT
	**B2M (Reference gene)**	AGATGAGTATGCCTGCCGTGT	TGCGACATCTTCAAACCTCCAT

Abbreviations: *STL* Sperm Telomere Length, *AMPK* AMP-activated Protein Kinase, *ULK1* Unc-51-Like Kinase 1, *BCL2* B-cell Lymphoma 2, *BAX* BCL2-Associated X, *B2M* Beta-2-Microglobulin

### Analysis of mRNA expression by qRT-PCR

The remaining washed samples were incubated on ice for 15 min in a somatic cell lysis solution to eliminate any somatic cell contaminations. Total RNA extraction and cDNA synthesis were carried out using RiboEx™ total RNA isolation solution (GeneAll Biotechnology, South Korea) and cDNA Synthesis Kit (Yekta-Tajhiz, Tehran, Iran), respectively. Furthermore, RealQ plus 2 × Master Mix Green (Ampliqon, Denmark) was utilized for qRT-PCR. For designing primer pairs for the target (Beclin1, AMPKa1, ULK1, BAX, and BCL2) and reference (Beta-2-Microglobulin (B2M)) genes, the NCBI Primer Blast tool was employed (Table 1). The mean cycle threshold (CT) of each target gene was normalized against B2M. Finally, the fold change in mRNA levels was computed utilizing the 2^(−ΔΔct)^ formula.

### Statistical analysis

GraphPad Prism v9.0.0 (GraphPad Software Inc, CA, USA) was utilized to conduct all statistical analyses. The data were reported as mean ± standard error of the mean (SEM). The Kolmogorov–Smirnov test was performed to determine the Gaussian distribution of the data. The independent sample t-test with Welch’s correction was conducted for assessing between-group differences. Pearson’s correlation coefficient was used to examine the correlations between STL and selected parameters. *p* < 0.05 was regarded to be statistically significant.

## Results

### Comparison of age, BMI, and conventional semen parameters between study groups

According to our results, there was no difference in the mean age between groups (*p* > 0.05). The mean BMI was significantly higher in the group with obesity than in the normal-weight group (31.36 ± 0.1726 vs 22.87 ± 0.2714, *p* < 0.0001). In the group with obesity, the mean values of semen volume, total sperm count, and progressive motility were all significantly lower than in the normal-weight group (*p* = 0.0074, 0.0407, and 0.0405, respectively). Other parameters, including the sperm concentration, total motility, non-progressive motility, and percentage of normal sperm morphology, were not statistically different across the groups (*p* > 0.05) (please see Table 2). In the normal-weight group, there was only one case of oligozoospermia and no cases of asthenozoospermia or teratozoospermia, In the group with obesity, there were one case of astheno-teratozoospermia, eight cases of teratozoospermia, and no cases of oligozoospermia or asthenozoospermia. Note that all semen samples were collected after 3–5 days of sexual abstinence. All mentioned parameters with their exact p values are reported in Table 2.

**Table 2:** Comparison of male age, body mass index, and sperm parameters between study groups

**Parameters**	**Normal-weight (n = 32)**	**Group with obesity (n = 32)**	**p-value**
**Age (year)**	37.63 ± 0.6201	37.72 ± 0.6901	0.9198
**BMI (kg/m**^**2**^**)**	22.87 ± 0.2714	31.36 ± 0.1726^a^	< 0.0001^****^
**Semen volume (ml)**	2.869 ± 0.1707	2.294 ± 0.1166^a^	0.0074^**^
**Sperm concentration (10**^**6**^**/ml)**	57.25 ± 2.301	60.59 ± 1.911	0.2680
**Total sperm count (10**^**6**^**/ejaculate)**	162.3 ± 9.632	137.4 ± 6.957^a^	0.0407^*^
**Progressive motility (%)**	44.44 ± 1.140	40.47 ± 1.511^a^	0.0405^*^
**Non-progressive motility (%)**	11.31 ± 0.7162	13.19 ± 0.7049	0.0668
**Total motility (%)**	55.75 ± 1.261	53.66 ± 1.424	0.2753
**Normal morphology (%)**	10.38 ± 0.8762	9.391 ± 0.9250	0.4427

All data are presented as Mean ± SEM. The independent sample t-test with Welch’s correction was conducted for the assessment of differences between the study groups. *P < 0.05, ** *P* < 0.01, *** *P* < 0.001, and **** *P* < 0.0001

In the group with obesity, only the mean values of semen volume, total sperm count, and progressive motility were significantly lower than in the normal-weight group (*p* = 0.0074, 0.0407, and 0.0405, respectively)

a Represents a significant difference between the groups with obesity and the normal-weight group

Abbreviations: *BMI* Body Mass Index

### Comparison of DFI, percentage of sperm with immature chromatin, and intracellular ROS levels between study groups

**Fig. 1:**
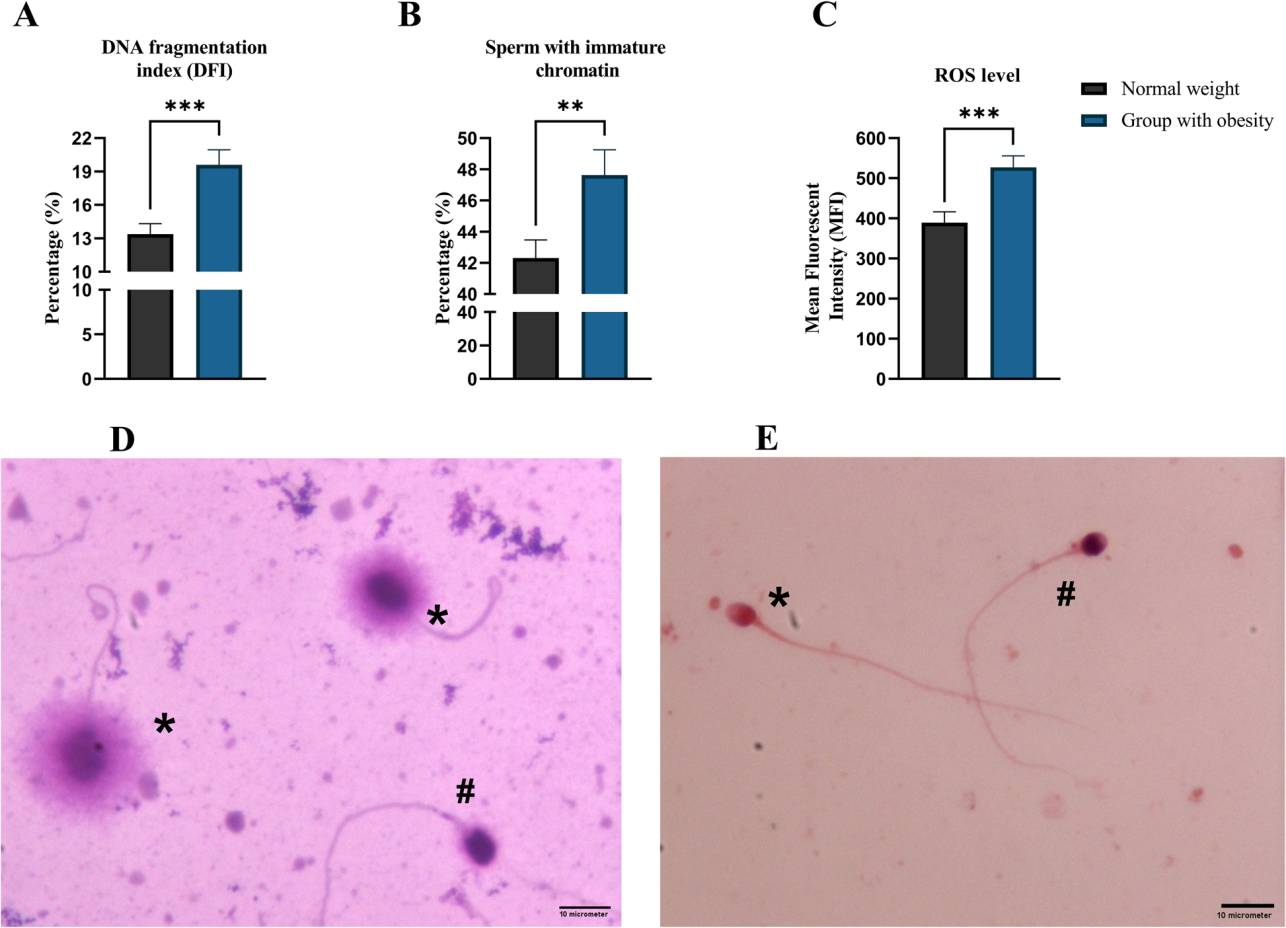
Comparison of DFI, percentage of sperm with immature chromatin, and ROS levels (MFI) between study groups. **A** Obesity resulted in significant increase in DFI (*p* = 0.0004) compared to normal-weight group. **B** Obesity also resulted in significant rise in percentage of sperm with immature chromatin (*p* = 0.0098) compared to normal-weight group. **C** Intracellular ROS level (*p* = 0.0009) was also significantly higher in group with obesity compared to normal-weight group. **A-C** All data are presented as Mean ± SEM. The independent sample t-test with Welch’s correction was conducted (*P < 0.05, ** *P* < 0.01, and *** *P* < 0.001). **D** Photomicrograph of the SCD test for assessing sperm DFI, 100 × objective. Sperm with fragmented DNA (#), Normal healthy sperm (*). **E** Photomicrograph of the AB staining for assessing sperm chromatin maturation, 100 × objective. Sperm with immature chromatin (#), Normal healthy sperm (*). Abbreviations: *ROS* Reactive Oxygen Species, *MFI* Mean Fluorescence Intensity, *DFI* DNA Fragmentation Index, *SCD* Sperm Chromatin Dispersion, *AB* Aniline Blue

As indicated in Fig. 1, there was a considerable increase in DFI in the group with obesity compared to the normal-weight group (*p* = 0.0004). Furthermore, the percentage of sperm with immature chromatin was found to be statistically higher in the group with obesity compared to the normal-weight group (*p* = 0.0098). In terms of intracellular ROS levels, the group with obesity showed dramatically higher ROS levels than the other group (*p* = 0.0009).

### Comparison of sperm viability status (percentages of live, apoptotic, and necrotic sperm) between study groups

**Fig. 2:**
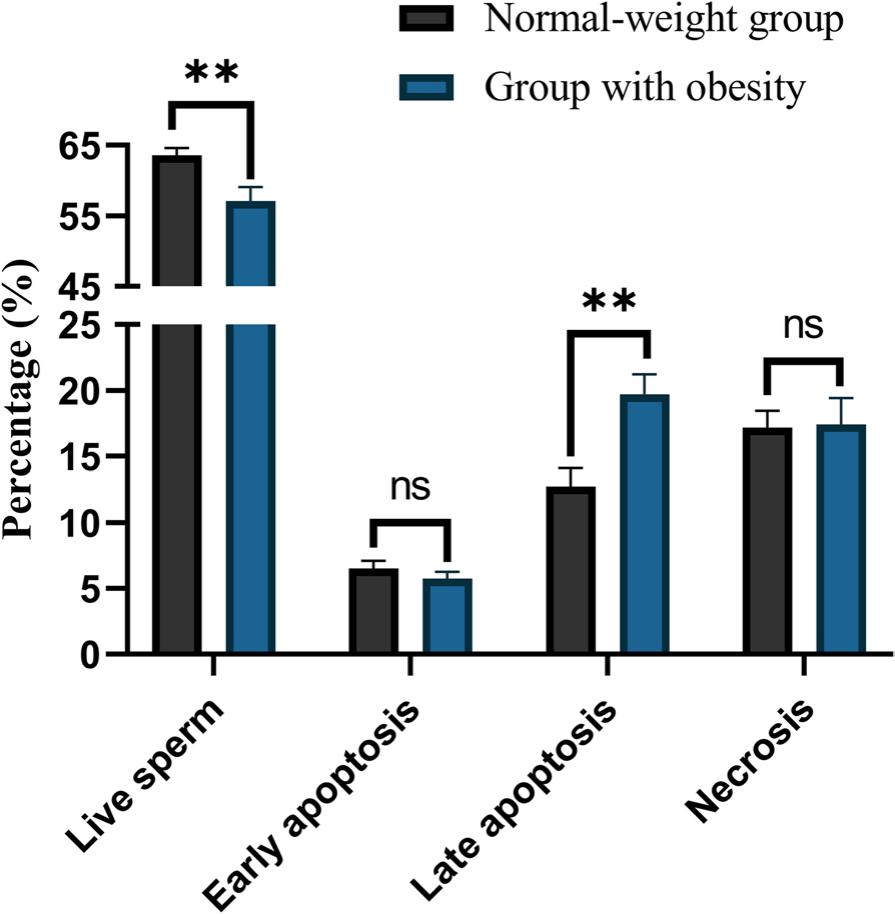
Comparison of sperm viability status between study groups. A substantial reduction in sperm viability (% of live sperm) was recorded in the group with obesity compared to the other group (*p* = 0.0059). Furthermore, we observed a significant increase in the percentage of late apoptotic sperm in the group with obesity compared to the other group (*p* = 0.0011). There were no significant differences between groups in terms of early apoptosis and necrosis (*p* > 0.05). All data are presented as Mean ± SEM. The independent sample t-test with Welch’s correction was conducted (*P < 0.05, ** *P* < 0.01, and *** *P* < 0.001)*.* Abbreviations: *ns* not significant

**Fig. 3:**
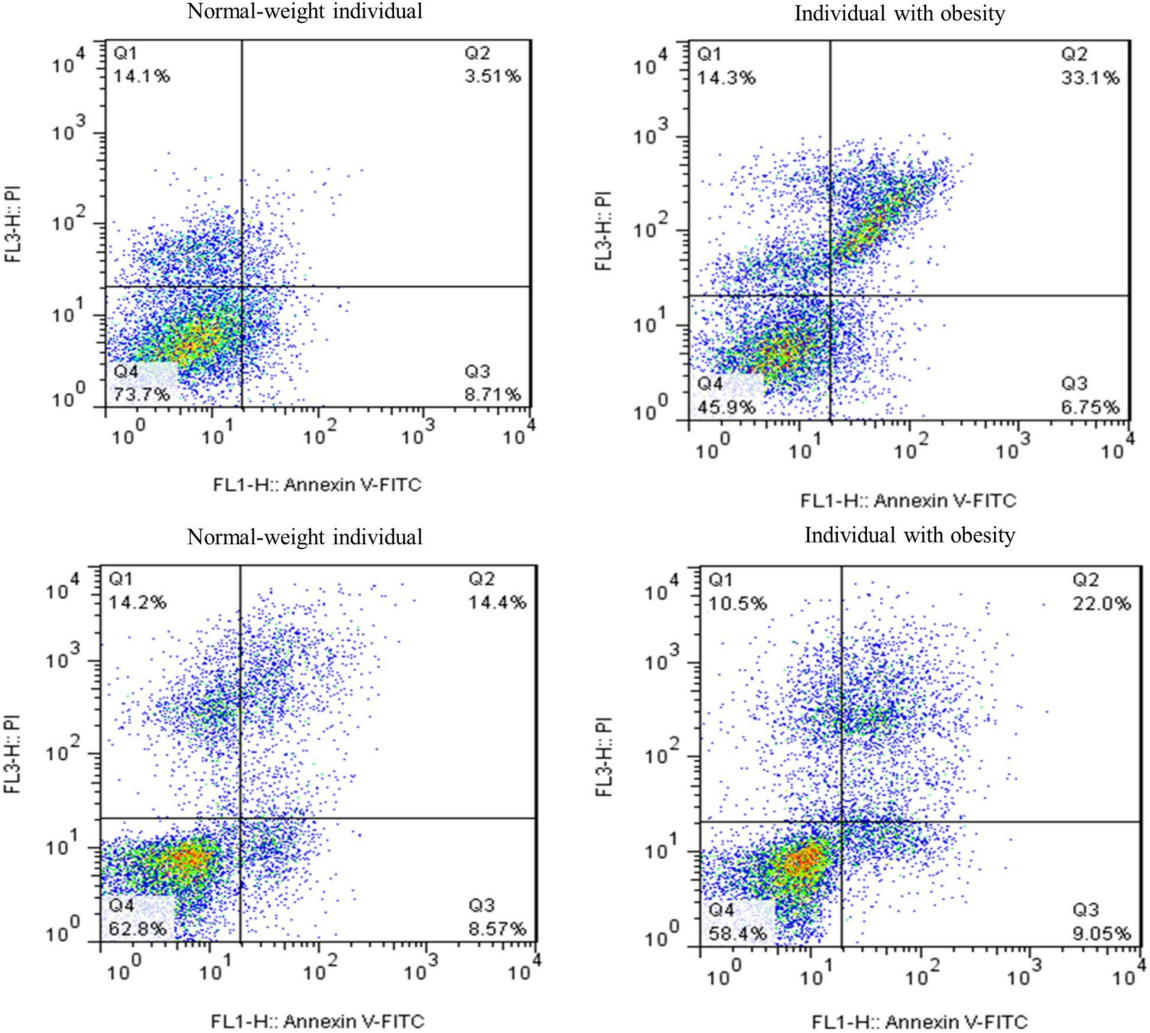
Assessing viability and apoptotic changes in sperm samples from individuals in the study groups utilizing annexin V-FITC/PI staining and flowcytometry. This figure describes the assessment of viability and apoptotic alterations in normal-weight individuals, as well as individuals with obesity. The Annexin V-FITC assay is based on Annexin V coupled with the fluorescent dye, FITC, as a probe for the detection of phosphatidylserine in the outer layer of the apoptotic cell membrane. PI is used to assess permeability of cell membranes. (Q1: Necrosis (%), Q2: Late Apoptosis (%), Q3: Early Apoptosis (%), and Q4: Live Cells (%)). Abbreviations: *PI*; Propidium Iodide, *FITC* Fluorescein isothiocyanate

Our data demonstrated a substantial reduction in sperm viability (% of live sperm) in the group with obesity compared to the other group (*p* = 0.0059). Furthermore, we observed a significant rise in the percentage of late apoptotic sperm in the group with obesity compared to the other group (*p* = 0.0011). There were no significant differences between groups in terms of early apoptosis and necrosis (*p* > 0.05). All mentioned parameters are presented in Figs. 2 and 3.

### Comparison of relative STL, and mRNA expression of Beclin1, AMPKa1, ULK1, BAX, and BCL2 between study groups

**Fig. 4:**
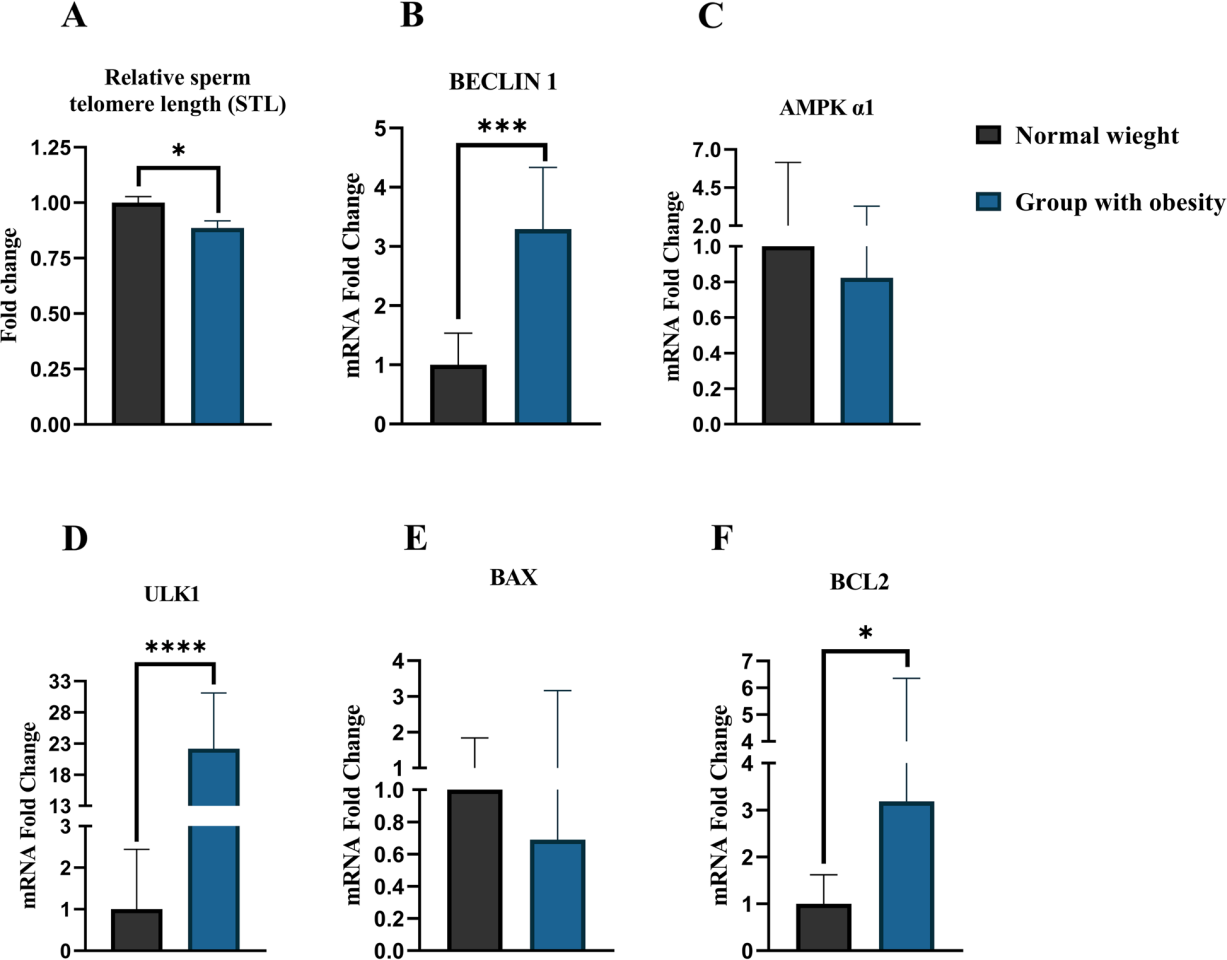
Comparison of relative STL, and autophagy-related mRNAs expression between study groups. **A** Obesity is associated with significant telomere shortening (*p* = 0.0117). **B-F** Comparison of relative mRNA expression between study groups showing significant upregulation of Beclin1 (*p* = 0.0002) (**B**), ULK1 (*p* < 0.0001) (**D**), and BCL2 (*p* = 0.0101) (**F**) in the group with obesity. AMPKa1 (*p* = 0.7892) (**C**) and BAX (*p* = 0.4463) (**E**) expression levels did not vary significantly across study groups. All data are presented as Mean ± SEM. The independent sample t-test with Welch’s correction was conducted (*P < 0.05, ** *P* < 0.01, and *** *P* < 0.001). Abbreviations: *STL* Sperm Telomere Length, *AMPK* AMP-activated Protein Kinase, *ULK1* Unc-51-Like Kinase 1, *BCL2* B-cell Lymphoma 2, *BAX* BCL2-Associated X

As illustrated in Fig. 4, there was a statistically significant reduction in relative STL in the group with obesity compared to the normal-weight group (*p* = 0.0117). Considering mRNA expression, Beclin1 (*p *= 0.0002), ULK1 (*p* < 0.0001), and BCL2 (*p* = 0.0101) were significantly upregulated in the group with obesity compared to the normal-weight group. Furthermore, no significant differences were found in the mRNA expression of AMPKa1 and BAX between the two groups. (*p* > 0.05).

### Correlation of relative STL with age, BMI, DFI, percentage of sperm with immature chromatin, and intracellular ROS levels in study groups

**Fig. 5:**
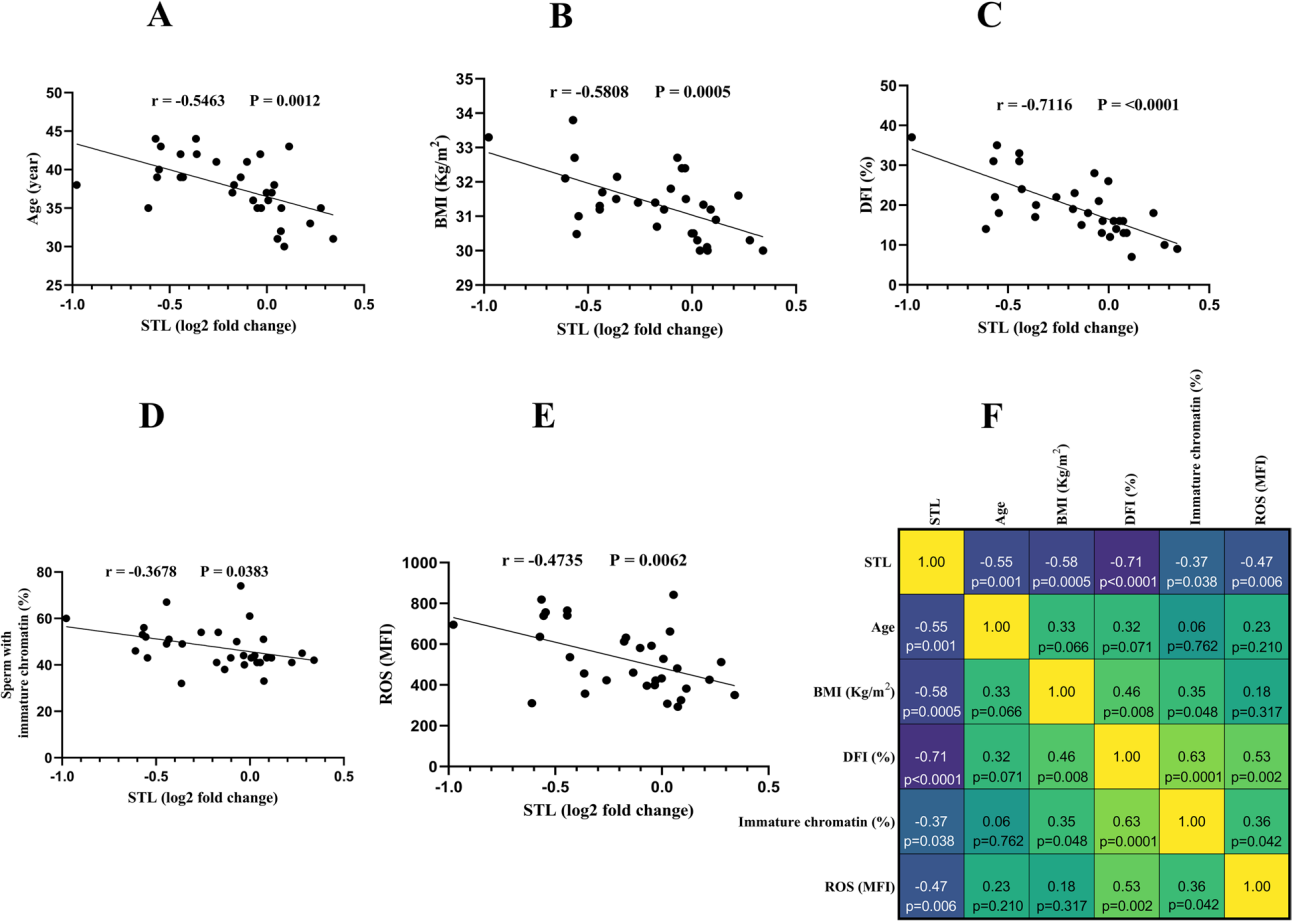
Correlation of relative STL with age, BMI, DFI, percentage of sperm with immature chromatin, and intracellular ROS levels in patients with obesity. **A** STL was negatively correlated with age (*P* = 0.0012) in patients with obesity. **B** STL was negatively correlated with BMI (*P* = 0.0005) in patients with obesity. **C** STL was negatively correlated with DFI (*P* < 0.0001) in patients with obesity **D** STL was negatively correlated with percentage of sperm with immature chromatin (*P* = 0.0383) in patients with obesity. **D** STL was negatively correlated with intracellular ROS levels (*P* = 0.0062) in patients with obesity. **F** The heat map representation of the correlation matrix with the precise r and p values for each of the parameters. Pearson’s correlation coefficient was used to examine the correlations between STL and selected parameters. *p* < 0.05 was regarded to be statistically significant. Abbreviations: *STL* Sperm Telomere Length, *BMI* Body Mass Index, *DFI* DNA Fragmentation Index, *ROS* Reactive Oxygen Species, *MFI* Mean Fluorescence Intensity

**Fig. 6:**
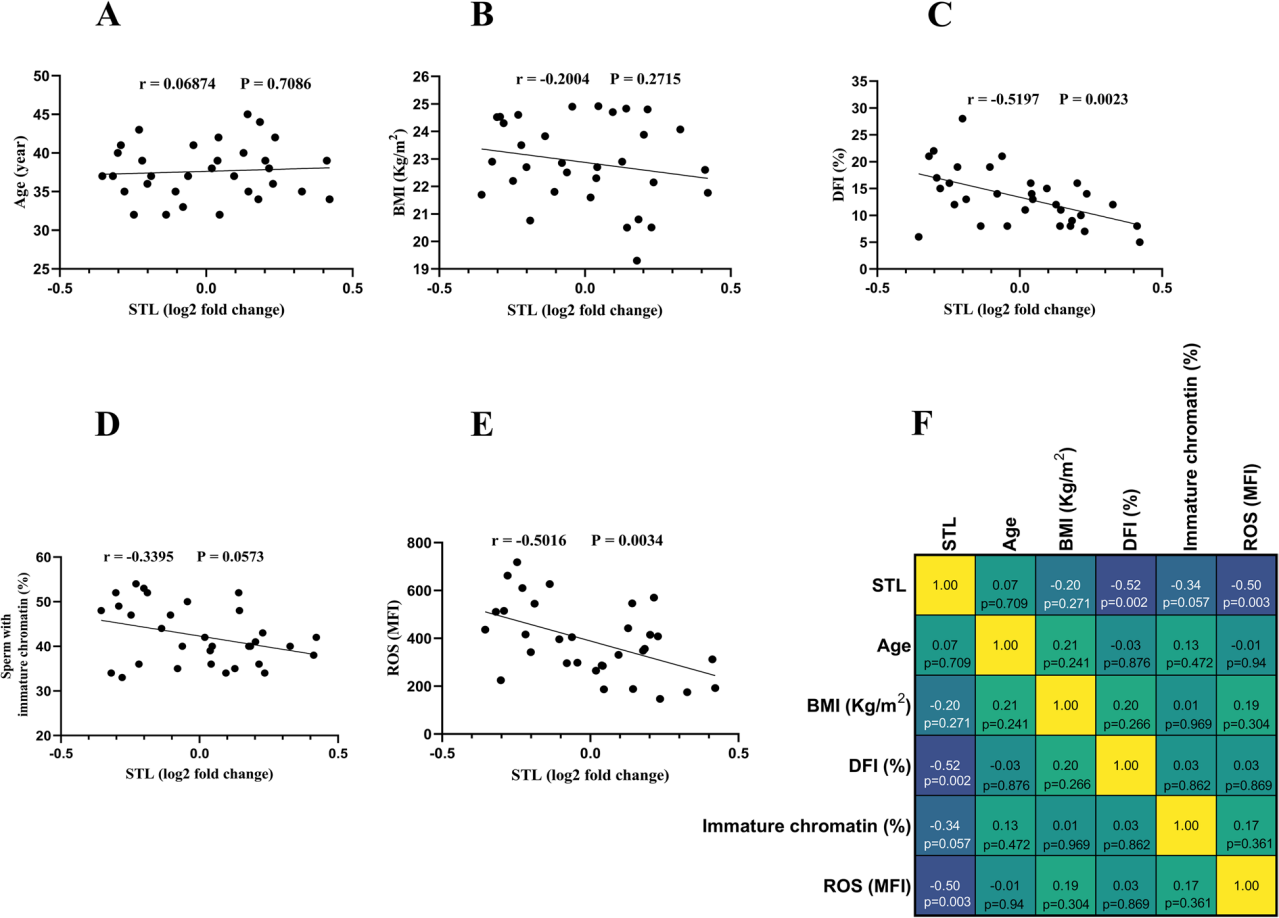
Correlation of relative STL with age, BMI, DFI, percentage of sperm with immature chromatin, and intracellular ROS levels in normal-weight patients. **A** There was no correlation between relative STL and age (*P* = 0.7086). **B** There was no correlation between relative STL and BMI (*P* = 0.2715). **C** STL was negatively correlated with DFI (*P* = 0.0023). **D** There was no correlation between relative STL and percentage of sperm with immature chromatin (*P* = 0.0573). **E** STL was negatively correlated with intracellular ROS levels (*P* = 0.0034). **F** The heat map representation of the correlation matrix with the precise r and p values for each of the parameters. Pearson’s correlation coefficient was used to examine the correlations between STL and selected parameters. *p* < 0.05 was regarded to be statistically significant. Abbreviations: *STL* Sperm Telomere Length, *BMI* Body Mass Index, *DFI* DNA Fragmentation Index, *ROS* Reactive Oxygen Species, *MFI* Mean Fluorescence Intensity

As presented in Fig. 5, the relative STL was negatively correlated with age (*P* = 0.0012), BMI (*P* = 0.0005), DFI (*P* < 0.0001), percentage of sperm with immature chromatin (*P* = 0.0383), and intracellular ROS levels (*P* = 0.0062) in patients with obesity. In the normal-weight group (Fig. 6) the relative STL was negatively correlated with DFI (*P* = 0.0023), and intracellular ROS levels (*P* = 0.0034), while no correlations were found between the relative STL and age (*P* = 0.7086), BMI (*P* = 0.2715), and percentage of sperm with immature chromatin (*P* = 0.0573).

## Discussion

It has long been considered that male fertility is negatively affected by obesity [4]. Despite major progresses in the field, the exact mechanisms and implications of this phenomenon on male fertility are yet to be understood. Accordingly, this research explored the association between obesity and male fertility from a new perspective.

In terms of semen parameters, we observed that obesity is adversely associated with total sperm count, semen volume, and progressive motility (Table 2). There are a number of studies that support these results [28–30]. As presented in previous studies [31, 32], we also observed that obesity leads to increased sperm DNA fragmentation, percentage of sperm with immature chromatin, and intracellular ROS levels (Fig. 1). It is known that sperm DNA integrity plays a critical role in male fertility potential, and any kind of damage that compromises its integrity can have a detrimental impact on male fertility and even embryonic development [31]. The process of sperm chromatin maturation is a naturally conserved phenomenon that ensures the integrity of sperm DNA. Histone-protamine exchange is a critical step in this process, which leads to nuclear condensation and so safeguards DNA from damage [31]. In the case of obesity, ROS plays a pivotal part in the development of OS, as its generation dramatically rises in this scenario [31, 33]. In addition to its detrimental effects on lipids and proteins, OS affects spermatozoa by inducing single- and double-strand DNA breakage [34]. In light of this, it is possible that sperm DNA integrity can be compromised far more severely whenever sperm chromatin maturation is disrupted. Here, we also found that obesity results in accelerated apoptosis, thus reducing sperm viability (Figs. 2 and 3). These findings of the adverse association between obesity and sperm viability are supported by other studies [28, 35]. We also observed that the percentages of spermatozoa with early apoptosis and necrosis are not different between patients with obesity and normal weight individuals, and for the first time we showed that lower sperm viability in patients with obesity is associated with higher percentages of late apoptosis.

In this study, we addressed the gaps in our understanding of obesity-related STL alterations. We observed that obesity is associated with a lower value of relative STL, resulting in sperm telomere shortening (Fig. 4A). This finding is in line with another study with a large sample size [13]. In the mentioned study, they recruited patients from infertile couples undergoing their first fresh IVF cycle with male BMI more than 28 (which includes overweight patients), whereas we recruited patients with obesity (BMI ≥ 30 kg/m^2^ according to WHO) from the general population and not merely the infertile category. As such, our patient population was entirely different. For the first time, we also demonstrated a significant negative correlation between relative STL and age, BMI, DFI, percentage of sperm with immature chromatin, and intracellular ROS levels in patients with obesity (Fig. 5). Telomere shortening in individuals with obesity may not be a direct consequence of obesity, but rather a multifactorial mechanism involving confounding variables that should be considered. One of the key mechanisms may be obesity-related OS and elevated ROS levels which contribute to telomere shortening. The effects of OS on telomere length are supported by studies indicating that conditions with elevated ROS levels (such as smoking and diabetes) can affect telomere length and result in telomere shortening [36–39]. In normal-weight individuals, we showed for the first time that relative STL was only negatively correlated with DFI and intracellular ROS levels, but not with age, BMI, or the percentage of sperm with immature chromatin (Fig. 6). These findings are interesting since they show a difference between two groups in terms of the correlation between relative STL and the factors indicated. As previously discussed, a DNA–protein structure called a telomere protects chromosomal ends against disintegration and fusion. Shortening of telomeric DNA occurs with each cell division, which gradually results in cellular senescence [8, 40]. Maintaining normal telomere length is crucial for keeping DNA integrity in cells [10, 12]. It is now known that the telomere can mediate the effect of factors associated with cellular senescence (ROS, and OS) on cells by affecting the expression of genes tightly related to apoptosis and cell death [40]. In addition, components of telomerase, an enzyme maintaining telomere length, are known to be abundantly expressed in human testis tissue, indicating the importance of telomere length maintenance during spermatogenesis [40]. Thus, sperm telomere shortening in patients with obesity has a significant association with decreased sperm DNA integrity (Fig. 5), and may be even their fertility potential. Despite these findings regarding STL and its correlation with sperm DNA integrity, still more detailed investigations with far larger sample sizes and on different patients are required before we can fully determine whether it can be implemented in clinical settings or not.

Here, for the first time, we evaluated changes in autophagy-related genes (AMPKa1, Beclin1, ULK1, BAX, and BCL2) mRNA expression in spermatozoa from patients with obesity. We reported significant up-regulation in mRNA expression of Beclin1, ULK1, and BCL2 in the patients with obesity (Fig. 4B-F). These genes are intimately linked to the autophagic process [17, 18, 41]. Autophagy is active in spermatozoa and has a role in cell survival and motility [16]. Furthermore, autophagy can be excessively activated through diet-induced obesity in mouse models [15]. Notably, autophagy can be activated in cells in response to ROS, an upstream autophagy-inducer, to maintain cellular homeostasis and viability, while excessive autophagy may actually lead to cell death [17, 18, 41]. Our data suggest that obesity is associated with considerable upregulation of autophagy-promoting genes in sperm, such as Beclin1 and ULK1. Additionally, BCL2, a negative regulator of Beclin1, was upregulated. As previously stated, BCL2 plays a critical part in cell survival by inhibiting apoptosis and modulating autophagy [18, 42]. In summary, the mRNA expression of genes involved in autophagy was dysregulated in the spermatozoa of a patient with obesity. Note that in order to generalize these results, further research including more patients is absolutely essential.

Based on what we discussed so far, OS combined with dysregulated autophagy-related gene expression, sperm telomere shortening, and subsequent DNA damage can be the possible reasons for reduced sperm quality in association with obesity.

## Limitations of the study

This study had some limitations that should be mentioned. First, a larger sample size would be significantly superior and provide stronger evidence. Although we employed a statistical approach to guarantee that our sample size was sufficient, we encourage researchers in the field to employ a far larger sample size so that we can generalize the current study's findings, especially molecular results. We also suggest assessing the expression of the aforementioned genes at the protein levels to have a brighter insight into the autophagic flux and how it changes in the case of obesity.

## Conclusions

Based on the data from the present study, obesity may be associated with lower total sperm count, semen volume, progressive motility, increased DFI, and a higher intracellular ROS level. Furthermore, obesity is possibly associated with sperm telomere shortening and aberrant autophagy-related genes mRNA expression. For the first time, we reported that STL is negatively correlated with age, BMI, DFI, percentage of sperm with immature chromatin, and intracellular ROS levels in patients with obesity, but only with DFI and ROS in normal-weight patients. We also observed that lower sperm viability in individuals with obesity may be attributed to a higher proportion of late-apoptotic sperm, rather than early apoptosis or necrosis. This study may open an avenue for improving the knowledge about the association between obesity and male fertility status, but further investigations are absolutely required.

## Data Availability

All data are available upon reasonable request.

## Abbreviations

WHO: World Health Organization
BMI: Body Mass Index
OS: Oxidative Stress
ROS: Reactive Oxygen Species
STL: Sperm Telomere Length
AMPK: AMP-activated Protein Kinase
mTORC1: Mammalian Target of Rapamycin Complex 1
ULK1: Unc-51-Like Kinase 1
VPS34: Vacuolar Protein Sorting 34
BCL2: B-cell Lymphoma 2
BAX: BCL2-Associated X
DFI: DNA Fragmentation Index
SCD: Sperm Chromatin Dispersion
DCFH-DA: 2'-7'Dichlorofluorescin Diacetate
qRT-PCR: Quantitative Qeal-Time PCR
AB: Aniline Blue
PBS: Phosphate Buffer Saline
PI: Propidium Iodide
MFI: Mean Fluorescent Intensity
B2M: Beta-2-Microglobulin
CT: Cycle Threshold
SEM: Standard Error of the Mean

## References

[1] Blüher M (2019). Obesity: global epidemiology and pathogenesis. Nat Rev Endocrinol.

[2] WHO. Tackling NCDs:'best buys' and other recommended interventions for the prevention and control of noncommunicable diseases. 2017. https://apps.who.int/iris/handle/10665/259232.

[3] WHO. World Health Organization Global Health Observatory (GHO) Data. Obesity and Overweight. 2016. https://apps.who.int/gho/data/node.main.A896?lang=en.

[4] Leisegang K, Sengupta P, Agarwal A, Henkel R. Obesity and male infertility: Mechanisms and management. Andrologia. 2021; 53(1): e13617.DOI: 10.1111/and.13617. 10.1111/and.13617 32399992

[5] Chaudhuri GR, Das A, Kesh SB, Bhattacharya K, Dutta S, Sengupta P (2022). Obesity and male infertility: multifaceted reproductive disruption. Middle East Fertility Society Journal.

[6] Barati E, Nikzad H, Karimian M (2020). Oxidative stress and male infertility: current knowledge of pathophysiology and role of antioxidant therapy in disease management. Cell Mol Life Sci.

[7] Pearce KL, Hill A, Tremellen KP (2019). Obesity related metabolic endotoxemia is associated with oxidative stress and impaired sperm DNA integrity. Basic Clin Androl.

[8] Cheng F, Carroll L, Joglekar MV, Januszewski AS, Wong KK, Hardikar AA (2021). Diabetes, metabolic disease, and telomere length. Lancet Diabetes Endocrinol.

[9] Gentiluomo M, Luddi A, Cingolani A, Fornili M, Governini L, Lucenteforte E (2021). Telomere Length and Male Fertility. Int J Mol Sci.

[10] Amirzadegan M, Sadeghi N, Tavalaee M, Nasr-Esfahani MH. Analysis of leukocyte and sperm telomere length in oligozoospermic men. Andrologia. 2021; 53(10): e14204.DOI: 10.1111/and.14204. 10.1111/and.14204 34369610

[11] Khosravaniardakani S, Bokov DO, Mahmudiono T, Hashemi SS, Nikrad N, Rabieemotmaen S, et al. Obesity Accelerates Leukocyte Telomere Length Shortening in Apparently Healthy Adults: A Meta-Analysis. Frontiers in Nutrition. 2022; 9.DOI: 10.3389/fnut.2022.812846. 10.3389/fnut.2022.812846 PMC9199514 35719148

[12] Darmishonnejad Z, Zarei-Kheirabadi F, Tavalaee M, Zarei-Kheirabadi M, Zohrabi D, Nasr-Esfahani MH. Relationship between sperm telomere length and sperm quality in infertile men. Andrologia. 2020; 52(5): e13546.DOI: 10.1111/and.13546. 10.1111/and.13546 32189393

[13] Yang Q, Zhao F, Hu L, Bai R, Zhang N, Yao G (2016). Effect of paternal overweight or obesity on IVF treatment outcomes and the possible mechanisms involved. Sci Rep.

[14] Zhang Y, Sowers JR, Ren J (2018). Targeting autophagy in obesity: from pathophysiology to management. Nat Rev Endocrinol.

[15] Mu Y, Yan W-J, Yin T-L, Zhang Y, Li J, Yang J. Diet-induced obesity impairs spermatogenesis: a potential role for autophagy. Sci. Rep. 2017; 7: 43475-.DOI: 10.1038/srep43475. 10.1038/srep43475 PMC5343591 28276438

[16] Aparicio IM, Espino J, Bejarano I, Gallardo-Soler A, Campo ML, Salido GM (2016). Autophagy-related proteins are functionally active in human spermatozoa and may be involved in the regulation of cell survival and motility. Sci Rep.

[17] Doherty J, Baehrecke EH (2018). Life, death and autophagy. Nat Cell Biol.

[18] Fernández ÁF, Sebti S, Wei Y, Zou Z, Shi M, McMillan KL (2018). Disruption of the beclin 1–BCL2 autophagy regulatory complex promotes longevity in mice. Nature.

[19] Xu H-D, Qin Z-H. Beclin 1, Bcl-2 and Autophagy, in Autophagy: Biology and Diseases: Basic Science, Qin Z-H, Editor. Singapore: Springer Singapore; 2019. 109–26. 10.1007/978-981-15-0602-4_5. 10.1007/978-981-15-0602-4_5 31776982

[20] Charan J, Biswas T (2013). How to calculate sample size for different study designs in medical research?. Indian J Psychol Med.

[21] WHO. WHO laboratory manual for the examination and processing of human semen. 6th ed. Geneva, Switzerland: WHO Press; 2021. https://www.who.int/publications/i/item/9789240030787.

[22] Fernández JL, Muriel L, Rivero MT, Goyanes V, Vazquez R, Alvarez JG (2003). The Sperm Chromatin Dispersion Test: A Simple Method for the Determination of Sperm DNA Fragmentation. J Androl.

[23] Auger J, Mesbah M, Huber C, Dadoune JP (1990). Aniline blue staining as a marker of sperm chromatin defects associated with different semen characteristics discriminates between proven fertile and suspected infertile men. Int J Androl.

[24] Pourmasumi S, Khoradmehr A, Rahiminia T, Sabeti P, Talebi AR, Ghasemzadeh J (2019). Evaluation of Sperm Chromatin Integrity Using Aniline Blue and Toluidine Blue Staining in Infertile and Normozoospermic Men. J Reprod Infertil.

[25] Robles V, Riesco MF, Martínez-Vázquez JM, Valcarce DG. Flow Cytometry and Confocal Microscopy for ROS Evaluation in Fish and Human Spermatozoa, in Reactive Oxygen Species: Methods and Protocols, Espada J, Editor. New York, NY: Springer US; 2021. 93–102. 10.1007/978-1-0716-0896-8_8. 10.1007/978-1-0716-0896-8_8 32857349

[26] Cawthon RM. Telomere measurement by quantitative PCR. Nucleic Acids Res. 2002; 30(10): e47-e.DOI: 10.1093/nar/30.10.e47. 10.1093/nar/30.10.e47 PMC115301 12000852

[27] Rocca MS, Speltra E, Menegazzo M, Garolla A, Foresta C, Ferlin A (2016). Sperm telomere length as a parameter of sperm quality in normozoospermic men. Hum Reprod.

[28] Taha EA, Sayed SK, Gaber HD, Abdel Hafez HK, Ghandour N, Zahran A (2016). Does being overweight affect seminal variables in fertile men?. Reprod Biomed Online.

[29] Eisenberg ML, Kim S, Chen Z, Sundaram R, Schisterman EF, Buck Louis GM (2014). The relationship between male BMI and waist circumference on semen quality: data from the LIFE study. Hum Reprod.

[30] Wang S, Sun J, Wang J, Ping Z, Liu L. Does obesity based on body mass index affect semen quality?—A meta-analysis and systematic review from the general population rather than the infertile population. Andrologia. 2021; 53(7): e14099.DOI: 10.1111/and.14099. 10.1111/and.14099 34028074

[31] Panner Selvam MK, Ambar RF, Agarwal A, Henkel R. Etiologies of sperm DNA damage and its impact on male infertility. Andrologia. 2021; 53(1): e13706.DOI: 10.1111/and.13706. 10.1111/and.13706 32559347

[32] Agarwal A, Farkouh Aa, Parekh N, Zini A, Arafa M, Kandil H, et al. Sperm DNA Fragmentation: A Critical Assessment of Clinical Practice Guidelines. World J Mens Health. 2022; 40(1): 30–7.DOI: 10.5534/wjmh.210056. 10.5534/wjmh.210056 PMC8761233 33988000

[33] Chianese R, Pierantoni R. Mitochondrial Reactive Oxygen Species (ROS) Production Alters Sperm Quality. Antioxidants. 2021; 10(1).DOI: 10.3390/antiox10010092. 10.3390/antiox10010092 PMC7827812 33440836

[34] Sepidarkish M, Maleki-Hajiagha A, Maroufizadeh S, Rezaeinejad M, Almasi-Hashiani A, Razavi M (2020). The effect of body mass index on sperm DNA fragmentation: a systematic review and meta-analysis. Int J Obes.

[35] Salas-Huetos A, Maghsoumi-Norouzabad L, James ER, Carrell DT, Aston KI, Jenkins TG, et al. Male adiposity, sperm parameters and reproductive hormones: An updated systematic review and collaborative meta-analysis. Obes. Rev. 2021; 22(1): e13082.DOI: 10.1111/obr.13082. 10.1111/obr.13082 32705766

[36] Barnes RP, Fouquerel E, Opresko PL (2019). The impact of oxidative DNA damage and stress on telomere homeostasis. Mech Ageing Dev.

[37] Lin J, Epel E. Stress and telomere shortening: Insights from cellular mechanisms. Ageing Research Reviews. 2022; 73: 101507.DOI: 10.1016/j.arr.2021.101507. 10.1016/j.arr.2021.101507 PMC8920518 34736994

[38] Morlá M, Busquets X, Pons J, Sauleda J, MacNee W, Agustí AGN (2006). Telomere shortening in smokers with and without COPD. Eur Respir J.

[39] Tahamtan S, Tavalaee M, Izadi T, Barikrow N, Zakeri Z, Lockshin RA (2019). Reduced sperm telomere length in individuals with varicocele is associated with reduced genomic integrity. Sci Rep.

[40] Demanelis K, Jasmine F, Chen Lin S, Chernoff M, Tong L, Delgado D, et al. Determinants of telomere length across human tissues. Science. 2020; 369(6509): eaaz6876.DOI: 10.1126/science.aaz6876. 10.1126/science.aaz6876 PMC8108546 32913074

[41] Yang Y, Klionsky DJ (2020). Autophagy and disease: unanswered questions. Cell Death Differ.

[42] Xu H-D, Wu D, Gu J-H, Ge J-B, Wu J-C, Han R, et al. The Pro-Survival Role of Autophagy Depends on Bcl-2 Under Nutrition Stress Conditions. PLoS One. 2013; 8(5): e63232.DOI: 10.1371/journal.pone.0063232. 10.1371/journal.pone.0063232 PMC3643928 23658815

